# Supplementary material for: The genome sequence of a chironomid fly, Chironomus tentans Fabricius, 1805
Source: Wellcome Open Res. 2025 Mar 17;10:133. [Version 1] doi: 10.12688/wellcomeopenres.23907.1 (PMC12038343; doi:10.12688/wellcomeopenres.23907.1)
Supplement: Peer review history

# Reviewer report: Reviewer response for version 1

**DOI:** 10.21956/wellcomeopenres.26375.r121556

**Published:** 2025-04-28

**Authors:** Terrence Sylvester

**Affiliations:** 1 University of memphis, memphis, USA

The authors present the genome assembly of a chironomid fly, *Chironomus tentans*. The genome is scaffolded up to the chromosome level and annotated. The genome assembly is of high quality with low base error and high kmer completeness. Interestingly the number of single copy othrologs that are present as complete copies are on the lower side. Other than that, I do not have significant remarks on the paper.

Are sufficient details of methods and materials provided to allow replication by others?

Yes

Is the rationale for creating the dataset(s) clearly described?

Yes

Are the datasets clearly presented in a useable and accessible format?

Yes

Are the protocols appropriate and is the work technically sound?

Yes

Reviewer Expertise:

Genomics

I confirm that I have read this submission and believe that I have an appropriate level of expertise to confirm that it is of an acceptable scientific standard.

# Reviewer report: Reviewer response for version 1

**DOI:** 10.21956/wellcomeopenres.26375.r121560

**Published:** 2025-04-16

**Authors:** Ann-Marie Waldvogel

**Affiliations:** 1 School of Life Sciences, Technical University of Munich, Munich, Germany

This report presents a novel genome assembly with chromosome resolution for the aquatic midge Chironomus tentans. The rationale for this genome project and description of the species is clear and appropriate for this purpose.

 The assembly is based on genomic DNA of one single individual, however, the sex of the individual is not mentioned. With regard to Figure 1, it seems to be a male (bushy antennae) but should be validated again. Reporting the sex of the assembled genome is relevant for future research on sex chromosome evolution - what might be one topic for which this genomic resource will be valuable.

 Data generation and assembly are based on state-of-the-art technology and methodology. This is also reflected in the high quality of the resulting assembly.

Are sufficient details of methods and materials provided to allow replication by others?

Yes

Is the rationale for creating the dataset(s) clearly described?

Yes

Are the datasets clearly presented in a useable and accessible format?

Yes

Are the protocols appropriate and is the work technically sound?

Yes

Reviewer Expertise:

Biodiversity genomics, limnology, ecology, population genomics

I confirm that I have read this submission and believe that I have an appropriate level of expertise to confirm that it is of an acceptable scientific standard, however I have significant reservations, as outlined above.

# Reviewer report: Reviewer response for version 1

**DOI:** 10.21956/wellcomeopenres.26375.r121562

**Published:** 2025-04-09

**Authors:** Claire Mérot

**Affiliations:** 1 Universite de Rennes, Rennes, Brittany, France

This is a short genome note describing the assembly of a chromosome-level reference for Chironomus tentans. 

 Background about the species is short but informative. Methods and protocols are well-explained and the report about the assembly is very clear. 

 This high-quality assembly will provide relevant resources for the community and this short note provides all necessary companion information.

Are sufficient details of methods and materials provided to allow replication by others?

Yes

Is the rationale for creating the dataset(s) clearly described?

Yes

Are the datasets clearly presented in a useable and accessible format?

Yes

Are the protocols appropriate and is the work technically sound?

Yes

Reviewer Expertise:

evolutionary genomics

I confirm that I have read this submission and believe that I have an appropriate level of expertise to confirm that it is of an acceptable scientific standard.
